# Supplementary material for: Allosteric regulation and crystallographic fragment screening of SARS-CoV-2 NSP15 endoribonuclease
Source: Nucleic Acids Res. 2023 Apr 28;51(10):5255–70. doi: 10.1093/nar/gkad314 (PMC10250223; doi:10.1093/nar/gkad314)
Supplement: gkad314_Supplemental_Files [file gkad314_supplemental_files.zip › Supplementary datasets.zip!/217/Refine_9/01-BUSTER/Cycle-4/shell.01/R_Rfree.html]

# Rxpct factors in resolution bins at cycle 0


|  |  |  |  |  |  |  |  |  |
| --- | --- | --- | --- | --- | --- | --- | --- | --- |
|  | **<d> (Å)**  Rxpct\_Work | Rxpct\_Free | Rxpct\_All | |  |  |  |  |
|  | 16.1098 | 0.3911 | 0.4424 | 0.3929 |  |
|  | 9.3010 | 0.2305 | 0.2724 | 0.2330 |  |
|  | 7.2045 | 0.1986 | 0.2668 | 0.2024 |  |
|  | 6.0889 | 0.1995 | 0.2708 | 0.2027 |  |
|  | 5.3699 | 0.1585 | 0.2458 | 0.1621 |  |
|  | 4.8573 | 0.1470 | 0.1929 | 0.1497 |  |
|  | 4.4681 | 0.1486 | 0.2157 | 0.1520 |  |
|  | 4.1595 | 0.1482 | 0.2148 | 0.1520 |  |
|  | 3.9072 | 0.1596 | 0.2385 | 0.1634 |  |
|  | 3.6958 | 0.1620 | 0.2292 | 0.1650 |  |
|  | 3.5155 | 0.1700 | 0.2026 | 0.1716 |  |
|  | 3.3591 | 0.1750 | 0.2354 | 0.1785 |  |
|  | 3.2220 | 0.1871 | 0.2242 | 0.1892 |  |
|  | 3.1003 | 0.1928 | 0.2148 | 0.1939 |  |
|  | 2.9915 | 0.1918 | 0.2464 | 0.1939 |  |
|  | 2.8934 | 0.2058 | 0.2456 | 0.2079 |  |
|  | 2.8044 | 0.2072 | 0.2301 | 0.2082 |  |
|  | 2.7231 | 0.2096 | 0.2729 | 0.2128 |  |
|  | 2.6484 | 0.2378 | 0.3011 | 0.2419 |  |
|  | 2.5796 | 0.2553 | 0.2851 | 0.2569 |  |
|  | **Overall** | 0.1867 | 0.2399 | 0.1894 |  |

# Rxpct factors in resolution bins at cycle 1


|  |  |  |  |  |  |  |  |  |
| --- | --- | --- | --- | --- | --- | --- | --- | --- |
|  | **<d> (Å)**  Rxpct\_Work | Rxpct\_Free | Rxpct\_All | |  |  |  |  |
|  | 16.1098 | 0.3917 | 0.4422 | 0.3934 |  |
|  | 9.3010 | 0.2301 | 0.2730 | 0.2326 |  |
|  | 7.2045 | 0.1988 | 0.2681 | 0.2026 |  |
|  | 6.0889 | 0.1997 | 0.2729 | 0.2030 |  |
|  | 5.3699 | 0.1585 | 0.2466 | 0.1621 |  |
|  | 4.8573 | 0.1469 | 0.1927 | 0.1495 |  |
|  | 4.4681 | 0.1486 | 0.2159 | 0.1520 |  |
|  | 4.1595 | 0.1481 | 0.2150 | 0.1520 |  |
|  | 3.9072 | 0.1595 | 0.2387 | 0.1634 |  |
|  | 3.6958 | 0.1620 | 0.2292 | 0.1650 |  |
|  | 3.5155 | 0.1699 | 0.2027 | 0.1715 |  |
|  | 3.3591 | 0.1750 | 0.2353 | 0.1785 |  |
|  | 3.2220 | 0.1871 | 0.2240 | 0.1892 |  |
|  | 3.1003 | 0.1927 | 0.2148 | 0.1938 |  |
|  | 2.9915 | 0.1918 | 0.2465 | 0.1939 |  |
|  | 2.8934 | 0.2058 | 0.2457 | 0.2079 |  |
|  | 2.8044 | 0.2071 | 0.2300 | 0.2081 |  |
|  | 2.7231 | 0.2095 | 0.2729 | 0.2127 |  |
|  | 2.6484 | 0.2376 | 0.3009 | 0.2417 |  |
|  | 2.5796 | 0.2551 | 0.2849 | 0.2568 |  |
|  | **Overall** | 0.1867 | 0.2402 | 0.1894 |  |

# Rxpct factors in resolution bins at cycle 2


|  |  |  |  |  |  |  |  |  |
| --- | --- | --- | --- | --- | --- | --- | --- | --- |
|  | **<d> (Å)**  Rxpct\_Work | Rxpct\_Free | Rxpct\_All | |  |  |  |  |
|  | 16.1098 | 0.3917 | 0.4423 | 0.3935 |  |
|  | 9.3010 | 0.2301 | 0.2731 | 0.2326 |  |
|  | 7.2045 | 0.1988 | 0.2682 | 0.2026 |  |
|  | 6.0889 | 0.1997 | 0.2731 | 0.2030 |  |
|  | 5.3699 | 0.1584 | 0.2464 | 0.1621 |  |
|  | 4.8573 | 0.1468 | 0.1929 | 0.1494 |  |
|  | 4.4681 | 0.1485 | 0.2159 | 0.1520 |  |
|  | 4.1595 | 0.1480 | 0.2149 | 0.1519 |  |
|  | 3.9072 | 0.1594 | 0.2386 | 0.1633 |  |
|  | 3.6958 | 0.1619 | 0.2294 | 0.1649 |  |
|  | 3.5155 | 0.1698 | 0.2026 | 0.1714 |  |
|  | 3.3591 | 0.1750 | 0.2351 | 0.1785 |  |
|  | 3.2220 | 0.1871 | 0.2239 | 0.1892 |  |
|  | 3.1003 | 0.1927 | 0.2151 | 0.1938 |  |
|  | 2.9915 | 0.1918 | 0.2468 | 0.1939 |  |
|  | 2.8934 | 0.2057 | 0.2460 | 0.2079 |  |
|  | 2.8044 | 0.2071 | 0.2299 | 0.2081 |  |
|  | 2.7231 | 0.2094 | 0.2729 | 0.2126 |  |
|  | 2.6484 | 0.2373 | 0.3010 | 0.2415 |  |
|  | 2.5796 | 0.2549 | 0.2849 | 0.2566 |  |
|  | **Overall** | 0.1866 | 0.2402 | 0.1893 |  |

# Rxpct factors in resolution bins at cycle 3


|  |  |  |  |  |  |  |  |  |
| --- | --- | --- | --- | --- | --- | --- | --- | --- |
|  | **<d> (Å)**  Rxpct\_Work | Rxpct\_Free | Rxpct\_All | |  |  |  |  |
|  | 16.1098 | 0.3865 | 0.4251 | 0.3878 |  |
|  | 9.3010 | 0.2306 | 0.2867 | 0.2340 |  |
|  | 7.2045 | 0.1966 | 0.2584 | 0.2000 |  |
|  | 6.0889 | 0.1993 | 0.2736 | 0.2026 |  |
|  | 5.3699 | 0.1572 | 0.2457 | 0.1608 |  |
|  | 4.8573 | 0.1458 | 0.1905 | 0.1483 |  |
|  | 4.4681 | 0.1470 | 0.2202 | 0.1507 |  |
|  | 4.1595 | 0.1457 | 0.2126 | 0.1496 |  |
|  | 3.9072 | 0.1561 | 0.2322 | 0.1599 |  |
|  | 3.6958 | 0.1600 | 0.2304 | 0.1631 |  |
|  | 3.5155 | 0.1677 | 0.2000 | 0.1693 |  |
|  | 3.3591 | 0.1734 | 0.2330 | 0.1768 |  |
|  | 3.2220 | 0.1865 | 0.2271 | 0.1887 |  |
|  | 3.1003 | 0.1918 | 0.2200 | 0.1932 |  |
|  | 2.9915 | 0.1935 | 0.2408 | 0.1953 |  |
|  | 2.8934 | 0.2060 | 0.2508 | 0.2084 |  |
|  | 2.8044 | 0.2071 | 0.2353 | 0.2083 |  |
|  | 2.7231 | 0.2087 | 0.2726 | 0.2119 |  |
|  | 2.6484 | 0.2390 | 0.2962 | 0.2427 |  |
|  | 2.5796 | 0.2562 | 0.2697 | 0.2569 |  |
|  | **Overall** | 0.1853 | 0.2397 | 0.1880 |  |
